# Effects of Psychological Interventions for Mental Health in Police Officers: A Systematic Review and Meta-Analysis

**DOI:** 10.3390/healthcare14081025

**Published:** 2026-04-13

**Authors:** Ga-In Lee, Jin-Hyuck Park

**Affiliations:** 1 Department of Rehabilitation Medicine, Good Neighbor Hospital, Cheoan 31070, Republic of Korea; syd1591@naver.com; 2 Department of Occupational Therapy, College of Medical Science, Soonchunhyang University, Asan 31538, Republic of Korea

**Keywords:** police officers, psychological intervention, mental health, posttraumatic stress disorder

## Abstract

Background/Objectives: Police officers are exposed to chronic occupational stress and traumatic events, placing them at increased risk for mental health problems. Previous meta-analyses have been limited by heterogeneous samples and methodological variability. This study evaluated the effectiveness of psychological interventions on mental health and posttraumatic stress disorder (PTSD) symptoms among police officers using randomized controlled trials (RCTs). Methods: A systematic search of PubMed, Embase, Web of Science, PsycINFO, and MEDLINE was conducted for studies published between January 2000 and September 2025. The search strategy utilized key terms including “police officers,” “psychological interventions,” “mental health,” and “randomized controlled trials”. Only RCTs involving police officers were included. Psychological interventions were compared with waitlist, usual-care, or active control conditions. General mental health outcomes (depression, anxiety, and stress) were analyzed as the primary outcome, and PTSD symptoms as a secondary outcome. Effect sizes were calculated as Hedges’s g using random-effects models. Subgroup, meta-regression, sensitivity, and publication bias analyses were conducted when appropriate. Results: Ten RCTs comprising 637 police officers met the inclusion criteria. Psychological interventions demonstrated moderate improvements in overall mental health (Hedges’s g = 0.516, 95% CI = 0.296–0.735, *p* < 0.001), albeit with substantial heterogeneity. Comparable effects were observed across waitlist and usual-care/active control comparisons. For PTSD symptoms, significant improvements were found only in comparisons with waitlist controls, whereas the overall pooled effect was not statistically significant. Meta-regression showed no dose–response relationship between total intervention hours and treatment effects. Sensitivity analyses confirmed result robustness. The certainty of evidence was rated as moderate for general mental health and low for PTSD symptoms, primarily due to substantial inconsistency and imprecision. Conclusions: These findings suggest that structured psychological programs show potential to confer added benefits for general mental health beyond routine wellness activities, although the certainty of the evidence is moderate to low. In contrast, the evidence for PTSD symptoms remains inconclusive, with effects failing to reach robust statistical significance. This underscores that preliminary individual-level intervention may be insufficient for trauma-specific symptoms, necessitating further research into specialized, trauma-focused approaches and the role of organizational determinants in enhancing intervention efficacy.

## 1. Introduction

Police officers are routinely exposed to recurrent traumatic events and chronic organizational stressors—such as shift work and administrative pressures—which synergistically elevate the risk of depression, anxiety, and posttraumatic stress disorder (PTSD) [1,2]. Current estimates suggest the prevalence of PTSD among police (5.8% to 19.6%) significantly exceeds that of the general population [3], underscoring an urgent need for evidence-based intervention strategies tailored to the unique demands of law enforcement [4].

To understand treatment receptivity in this population, it is essential to consider the link between trauma and dissociation. Dissociation acts as a peri-traumatic defense mechanism that while facilitating short-term focus, can create barriers to standard intervention by fragmenting trauma processing [5,6]. Consequently, addressing these complex psychological stressors requires integrative, broad-based approaches that move beyond simple symptom reduction to foster long-term psychological resilience [7,8]. Utilizing this perspective explains why a wide range of psychological intervention rather than a single specific modality was evaluated to capture the diverse mechanisms of recovery in police contexts.

The necessity of such integrative approaches is grounded in the fact that PTSD and general mental health indicators (e.g., depression, anxiety, stress) represent intertwined dimensions of occupational psychopathology [9]. Chronic organizational stressors often erode psychological resilience, increasing vulnerability to PTSD [10], while untreated trauma frequently generalizes into broader emotional distress [11]. Analyzing these outcomes together allows for a holistic evaluation of how interventions address both acute trauma responses and the underlying psychological baseline. This comparative approach is essential for identifying whether specific intervention yield uniform benefits across different clinical dimensions or if distinct recovery trajectories exist depending on the outcome measured [12].

Despite this emerging body of literature on psychological interventions including cognitive behavioral therapy (CBT) and mindfulness-based intervention (MBIs) [13,14], several methodological gaps persist. First, prior meta-analyses have often relied on heterogeneous first responder samples, failing to provide police-specific insights [15], or have lacked quantitative synthesis, precluding objective effect size estimation [16]. Third, even quantitative reviews have frequently restricted their scope to specific modalities (e.g., only MBIs) [17] or combined RCTs with quasi-experimental design, introducing substantial bias and heterogeneity [18]. Crucially, these previous works often overlook the unique socio-cultural milieu of law enforcement—characterized by a rigid warrior ethos and high perceived stigma—which can fundamentally alter the receptivity and stability of psychological interventions. This oversight suggests that universal intervention models may not account for the specific psychological constraints of policing, highlighting the need for a more theoretically grounded and police-specific analysis [19]. These limitations highlight the need for a rigorous and police-specific meta-analysis based on RCTs that differentiates control conditions and systematically examines explores sources of heterogeneity.

The present study addresses these gaps by providing a rigorous meta-analysis exclusively focused on RCTs within the unique socio-cultural milieu of law enforcement. By isolating the police-specific effect from broader first-responder data, this work offers a substantive theoretical advance in understanding how difference modalities perform within this high-risk profession. Specifically, this study: (1) utilizes subgroup analyses to distinguish between waitlist and active/usual-care controls to isolate specific therapeutic benefits from general supports effects; (2) disaggregates outcomes into general mental health and PTSD to identify distinct recovery trajectories across difference clinical dimensions; and (3) performs a comparative analysis across multiple modalities, including CBT and MBIs, to clarity which approaches yield the most robust benefits for police officers; and (4) employ meta-regression to examine whether intervention dose significantly moderates the observed effect sizes.

## 2. Materials and Methods

This systematic review and meta-analysis was conducted in strict accordance with the PRISMA 2020 guidelines Table S1 and prospectively registered with PROSPERO (ID: CRD420251177475). While the study followed the pre-specified protocol, an exploratory post hoc subgroup analysis was performed to further investigate the substantial heterogeneity identified during the primary analysis.

### 2.1. Search Strategy

The electronic databases were searched on 10 October 2025, using PubMed, Embase, Web of Science, PsycINFO, and MEDLINE to search for peer-reviewed articles published from January 2000 to September 2025 with no language restriction. The year 2000 was selected as the temporal cut-off because structured psychological interventions in their modern form (e.g., manualized CBT-based resilience training and standardized mindfulness) became predominantly established and evaluated using rigorous RCT designs from this period onwards. The search terms were combined with the AND operation in accordance with a previous study: (first responders OR police OR officer OR law enforcement) AND (cognitive behavioral therapy OR mindful OR psychological OR mental) AND (training OR intervention OR therapy OR education OR program) AND (depression OR anxiety OR stress OR posttraumatic stress disorder OR mental health) [10]. While keywords were focused on primary clinical outcomes, broader constructs such as “psychological distress,” “burnout,” or “well-being” were captured through the inclusion of the terms “mental,” “psychological,” and “mental health,” as the validated instruments used in the included studies are designed to measure these comprehensive psychological states.

To mitigate publication bias, we extended our search to clinical trial registries, including ClinicalTrials.gov and the ISRCTN registry, to identify unpublished or ongoing studies. A manual search of grey literature was performed via Google on 11 October 2025, using the pre-specified keywords. Furthermore, an updated search was conducted immediately prior to the final submission to capture the most recent literature; notably, no additional RCTs meeting the eligibility criteria were identified. The initial screening was independently performed by two reviewers based on titles and abstracts, followed by a meticulous full-text review of potentially relevant articles.

### 2.2. Study Selection Strategy

Studies were included based on the following eligibility criteria: (1) participants were active-duty police officers, recruits, or civilian personnel, regardless of baseline clinical status; (2) interventions consisted of psychological training aimed at enhancing mental health; (3) comparisons involved no-treatment, wait-list, usual-care, or active controls; (4) outcomes targeted general mental health (depression, anxiety, stress) or PTSD symptoms using validated measures; and (5) the study design was limited to randomized controlled trials (RCTs). We excluded adjunctive treatments where psychological effects could not be isolated, mixed-population studies without disaggregated police data, and trials with insufficient data for effect size calculation. Following duplicate removal, two independent reviewers (G.-I. Lee and J.-H. Park) executed a two-stage screening process (title/abstract followed by full-text). To ensure consistency, a random 10% subset was assessed jointly, with any discrepancies resolved through consensus or a third senior adjudicator. Inter-rater reliability, measured by Cohen’s Kappa, indicated near-perfect agreement (0.94 for screening; 0.92 for full-text review).

### 2.3. Data Extraction

Data were independently extracted by two reviewers using a standardized coding form. The following information was recorded: (1) participant characteristics, including sample size, mean age, and years of service; (2) intervention characteristics, such as the theoretical basis (e.g., CBT, mindfulness) and contents, number of sessions, and total duration (hours); (3) control group type (e.g., waitlist, usual-care, or active control); and (4) outcome data, specifically means, standard deviations (SDs), and sample sizes for both pre- and post-intervention assessments. In cases where data were reported as t-values, F-values, or *p*-values, these were converted to standardized mean differences. If essential data were missing, we contacted the corresponding authors to request the information. In cases where authors were unresponsive, missing SDs were handled according to the Cochrane Handbook guidelines. We derived missing SDs from other available statistics (e.g., confidence intervals, t-values) or, if necessary, imputed them using the mean SD from other trials within the same analysis that utilized the same measurement instrument. Studies with irrecoverable data were excluded to maintain the integrity of the synthesis. Any discrepancies in data extraction were resolved through discussion and consensus.

### 2.4. Intervention and Outcomes

Interventions included in this review were psychological intervention programs designed to enhance the mental health of police officers. These programs were based on various theoretical frameworks, including CBT, MBIs, and resilience enhancement models. The decision to provide an initial aggregate estimate across these diverse modalities is theoretically justified by their shared transdiagnostic objective: the enhancement of emotional regulation and cognitive coping to mitigate the unique occupational stressors of police officers [20]. Despite procedural differences, these interventions all target core mechanisms of occupational psychopathology—specifically, maladaptive cognitive appraisals and emotional dysregulation. This initial pooling establishes a global benchmark for psychological support in this field, providing a necessary baseline for our subsequent, more granular subgroup analyses [21].

The primary outcomes assessed were general mental health, operationalized as a composite measure of depression, anxiety, and stress. The aggregation of these symptoms into a single construct is grounded in the tripartite model of psychopathology, which posits that depression, anxiety, and stress share a core component of negative affectivity [22]. Furthermore, established instruments such as the Depression Anxiety Stress Scale-21 validated this integrated approach by demonstrating that these indicators collectively represent a robust measure of overall psychological distress in high-stress populations [23]. Depression and anxiety were evaluated using validated scales such as the Patient-Reported Outcomes Measurement Information System, the Beck Depression Inventory. Secondary outcomes included PTSD symptoms, captured using the Posttraumatic Stress Disorder Checklist for DSM-5 and the Structured Interview for PTST. All outcomes were evaluated using standardized assessment or self-report instruments.

### 2.5. Quality Assessment

The methodological quality of the included RCTs was evaluated using the Cochrane Risk of Bias tool (RoB 2) (August 2019 version) [24]. Two reviewers independently assessed five key domains: randomization process, deviations from intended interventions, missing outcome data, measurement of the outcome, and selection of the reported result. Studies were subsequently categorized as having ‘Low risk,’ ‘Some concerns,’ or ‘High risk’ of bias. Any discrepancies were resolved through consensus or consultation with a third reviewer. To ensure the robustness of our findings, the impact of bias was systematically addressed by conducting sensitivity analyses that excluded ‘High risk’ studies. Furthermore, publication bias was assessed via funnel plot asymmetry and Egger’s Test [25]. In cases of significant asymmetry, the Trim-and-Fill technique [26] was employed for adjustment. Notably, publication bias assessments were restricted to outcomes with at least ten studies to maintain adequate statistical power, as per established guidelines.

### 2.6. Statistical Analysis

Meta-analyses were performed using Comprehensive Meta-Analysis (CMA) software, version 3 (Biostat, Englewood, NJ, USA). Effect sizes were calculated as Hedges’s g based on the differences in change scores (mean change from baseline to post-intervention) between the intervention and control groups. This a small-sample bias-corrected standardized mean difference was interpreted using benchmarks of 0.20, 0.50, and 0.80 for small, moderate, and large effects, respectively [27]. Means, standard deviations, and sample sizes were extracted from each study to compute effect sizes; in cases where other statistics were reported, these were converted into Hedges’s g using established procedures. To ensure the independence of the data and avoid unit-of-analysis errors, each RCT contributed only one effect size to the primary meta-analysis. For studies reporting multiple outcomes for general mental health (e.g., both depression and stress), a composite effect size was calculated by averaging the Hedges’s g values of these outcomes. The variance for the composite effect size was computed assuming a within-study correlation (r) of 0.5 between outcomes, following established meta-analytic guidelines [28]. To determine the intervention effect sizes, both baseline (pre-intervention) and post-intervention data were extracted for all outcomes. This approach ensures that treatment effects are evaluated in the context of initial scores, thereby accounting for baseline comparability. However, follow-up assessments were excluded from the meta-analysis to maintain temporal consistency across the included trials and to prevent potential bias arising from the high variability in follow-up durations. Pooled effect size estimates were calculated only when at least two independent studies reported data for the same outcome.

Statistical heterogeneity was evaluated using the I^2^ statistic, which quantifies the proportion of observed variance attributable to real differences between studies rather than sampling error. Consistent with established benchmarks, I^2^ values of ≥50% were interpreted as indicating substantial and considerable heterogeneity, respectively [29]. To account for the inherent variability in intervention protocols and participant characteristics across the included RCTs, a random-effects model was employed for all syntheses to provide a more conservative and generalizable estimate of the pooled effect.

### 2.7. Subgroup Analysis Sensitivity Analysis

To investigate potential sources of heterogeneity, subgroup analyses were performed for both primary and secondary outcomes using categorical moderators. Specifically, we examined whether treatment effects varied by control condition (active/usual-care vs. passive/waitlist controls). Furthermore, to evaluate the robustness of our findings, we conducted sensitivity analyses by re-estimating the pooled effects after excluding trials with a ‘High risk’ of bias. This allowed us to determine the extent to which methodological quality influenced the overall estimates. Finally, publication bias was assessed via visual inspection of funnel plots and Egger’s regression intercept, with these tests restricted to outcomes represented by at least ten studies to ensure adequate statistical power.

### 2.8. Meta-Regression

To further investigate potential sources of heterogeneity, we conducted meta-regression analyses to determine whether treatment effects were moderated by intervention dose. Dose was operationalized as the total number of prescribed session hours for each intervention. This analytical approach enabled us to evaluate whether increased cumulative exposure to psychological training was associated with more robust improvements in mental health outcomes. Consistent with methodological recommendations, meta-regression analyses were performed only when at least ten studies were available for the outcomes of interest, as fewer studies yield insufficient statistical power and unstable parameter estimates [30]. Nevertheless, given the relatively small number of studies meeting this threshold, these analyses may still possess limited power to detect subtle moderator effects. Accordingly, the results of the meta-regression were treated as exploratory rather than definitive, and caution was exercised in their interpretation to avoid over-generalization.

### 2.9. Criteria for Assessing Certainty of Evidence

The certainty of evidence for the primary outcomes—general mental health and PTSD symptoms—was evaluated using the GRADE (Grading of Recommendations Assessment, Development and Evaluation) framework. Our assessment encompassed five critical domains: risk of bias, inconsistency, indirectness, imprecision, and publication bias. Following established standards [31], the certainty was classified into four levels: high, moderate, low, or very low. This evaluation was performed independently by two reviewers (G.-I. Lee and J.-H. Park), with any grading discrepancies resolved through consensus or consultation with a third senior adjudicator.

## 3. Results

### 3.1. Study Selection

**Figure 1:**
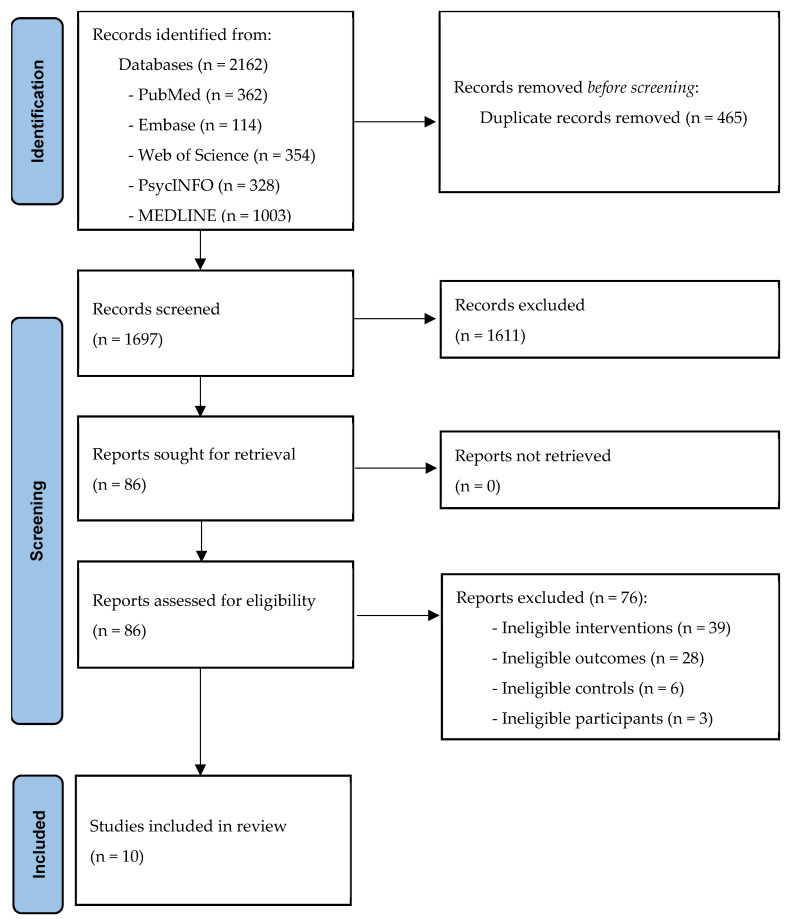
PRISMA flow of study selection process.

Figure 1 presents the PRISMA flow chart for the study selection process. Following the removal of duplicate records, a total of 1697 citations underwent initial screening based on titles and abstracts. From these, 86 potentially relevant articles were retrieved for a detailed full-text eligibility assessment. Ultimately, 10 independent studies met all inclusion criteria and were synthesized in this meta-analysis. Crucially, all 10 included studies were peer-reviewed articles, with no conference abstracts meeting the final criterial for quantitative synthesis. These studies provided complete data required for effect size calculation.

### 3.2. Study Characteristics

Table 1 presents the characteristics of the included studies. A total of 10 RCTs were included in this meta-analysis, comprising active-duty police officers across all studies. The combined sample consisted of 637 participants (Experimental group: *n* = 314; Control group: *n* = 323). Geographically, the studies showed a broad distribution, with four conducted in the USA, two in India, and one each in Australia, Brazil, the Netherlands, and Thailand. Reported mean ages ranged from 26.8 to 43.9 years, although several studies did not provide age information. Regarding the gender composition, the majority of participants were male, reflecting the typical demographic profile of law enforcement. Specifically, seven studies utilized mixed-gender samples with male representation ranging from 67.4% to 90.5%, while two studies focused exclusively on males and one study was dedicated solely to female officers.

Across the included trials, a diverse range of psychological interventions was implemented. To further investigate the sources of heterogeneity, these interventions were categorized into two primary modalities: (1) MBIs, such as Mindfulness-Based Resilience Training (MBRT) and Mindfulness-Based Stress Reduction (MBSR), which focus on non-judgmental awareness and physiological regulation; and (2) Non-MBIs, which encompass structured programs focusing on resilience enhancement, cognitive-behavioral strategies, coping skills training, and physiological stress management (e.g., biofeedback-assisted or imagery-based training). Intervention duration varied considerably, ranging from 3 to 20 sessions, with total program lengths spanning 1 to 30 h.

Control conditions differed across studies and included waitlist controls, usual care including psychoeducation, and in some cases active control conditions, such as critical incident simulation or stress management training.

A wide array of validated psychological outcome measures was used across studies. Depression and anxiety were measured using tools including the Patient-Reported Outcomes Measurement Information System-Depression (PROMIS-D), the Patient-Reported Outcomes Measurement Information System-Anxiety (PROMIS-A), the Beck Depression Inventory (BDI), the Hospital Anxiety and Depression (HADS), and the Symptom Checklist-90 (SCL-90). Stress was assessed using instruments such as the Perceived Stress Scale-10 (PSS-10), the Police Stress Questionnaire (PSQ), the Visual Analogue Scale (VAS), and the Occupational Stress Inventory (OSI). PTSD symptoms were captured using the Posttraumatic Stress Disorder Checklist for DSM-5 (PCL-5) and the Structured Interview for PTSD (SI-PTSD). Table 1 summarizes the detailed characteristics of the included studies.

### 3.3. Effect Size of Psychological Interventions on Mental Health

**Figure 2:**
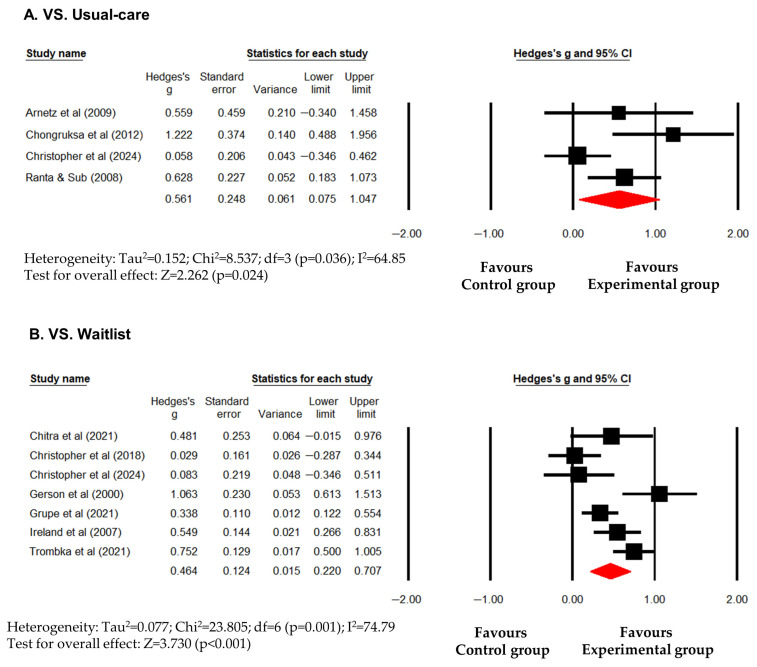
Forest plot showing effect sizes (Hedges’s g) of psychological interventions on mental health in police officers. (**A**) effect size compared to usual-care/active controls. (**B**) effect size compared to waitlist. Squares represent effect sizes with 95% confidential intervals for individual studies, while diamond indicate the pooled effect size. Arnetz et al. [32], Chongruksa et al. [34], Christopher et al. [35], Ranta & Sud [40], Chitra et al. [33], Christopher et al. [36], Christopher et al. [35], Gersons et al. [37], Grupe et al. [38], Ireland et al. [39], Trombka et al. [41].

**Figure 3:**
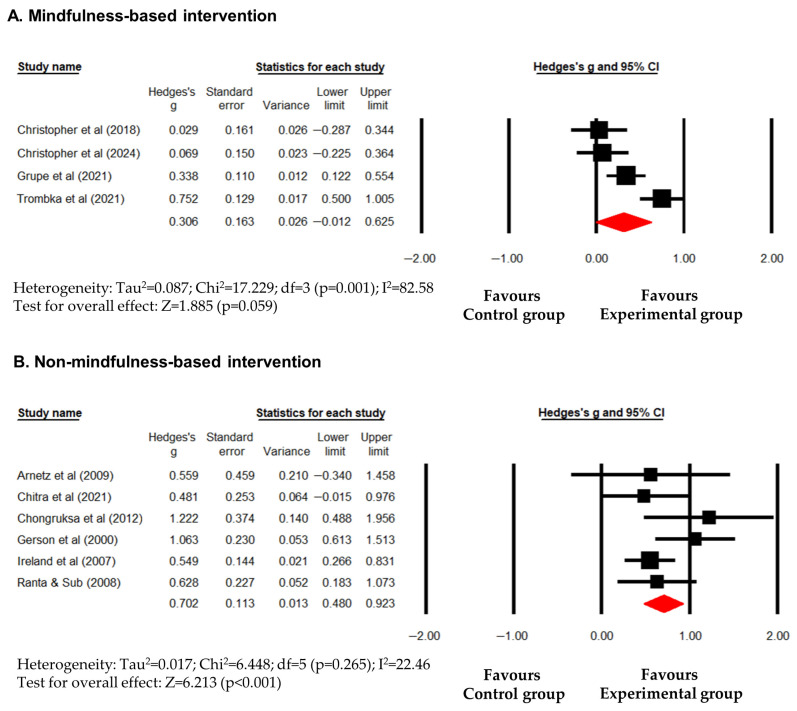
Forest plot showing effect sizes (Hedges’s g) of psychological interventions on mental health in police officers. (**A**) effect size of mindfulness-based interventions. (**B**) effect size of non-mindfulness-based interventions. Squares represent effect sizes with 95% confidential intervals for individual studies, while diamond indicate the pooled effect size. Arnetz et al. [32], Chitra et al. [33], Chongruksa et al. [34], Christopher et al. [35], Christopher et al. [36], Gersons et al. [37], Grupe et al. [38], Ireland et al. [39], Ranta & Sud [40], Trombka et al. [41].

Overall, psychological interventions showed a significant improvement in mental health (Hedges’s g (95% CI) = 0.516 (0.296 to 0.735); *p* < 0.001). However, this result was accompanied by significant heterogeneity (I^2^ = 72.16; *p* < 0.001), suggesting that the magnitude of the effect varied considerably across studies. Compared to usual-care/active controls, psychological interventions showed a significant improvement in mental health (Hedges’s g (95% CI) = 0.561 (0.075 to 1.047); *p* = 0.024). Significant heterogeneity was observed (I^2^ = 64.85; *p* = 0.036) (Figure 2A). Compared to waitlist controls, psychological interventions also significantly improved mental health (Hedges’s g (95% CI) = 0.464 (0.220 to 0.707); *p* < 0.001) with significant heterogeneity (I^2^ = 74.79; *p* = 0.001) (Figure 2B). While these findings suggest that a positive average trend, the high I^2^ values across all control types warrant caution, as the intervention impact was not uniform. To further explore the source of heterogeneity, an exploratory subgroup analysis was conducted by intervention type (MBIs vs. non-MBIs). MBIs showed a smaller, non-significant trend toward improvement (Hedges’s g (95% CI) = 0.306 (−0.012 to 0.625); *p* = 0.059) accompanied by substantial heterogeneity (I^2^ = 82.58%; *p* = 0.001) (Figure 3A), indicating highly inconsistent outcomes within MBIs. In contrast, non-MBIs (e.g., resilience training, CBT-based coping skills) provided relatively more stable and significant benefit (Hedges’s g (95% CI) = 0.702 (0.480 to 0.923); *p* < 0.001) with notably low heterogeneity (I^2^ = 22.46%; *p* = 0.265) (Figure 3B). These results suggest that the high overall heterogeneity was primarily driven by the variability within MBIs, whereas non-MBIs provided more stable and robust benefits for police officers’ general mental health. However, given the limited number of studies in these subgroups—particularly for MBIs—they should be viewed as exploratory, with the relatively more stable effect observed in non-MBIs providing a basis for further targeted research.

### 3.4. Effect Size of Psychological Interventions on PTSD Symptoms

**Figure 4:**
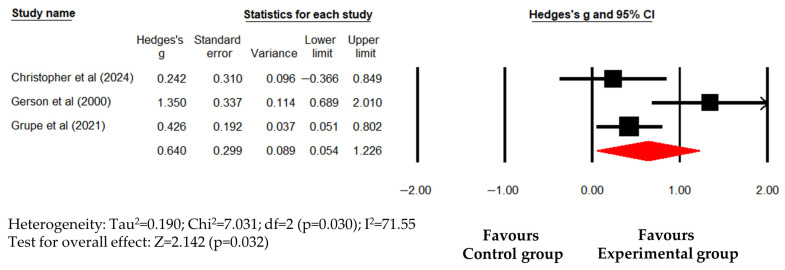
Forest plot showing effect sizes (Hedges’s g) of psychological interventions on PTSD symptoms in police officers compared to waitlist. Squares represent effect sizes with 95% confidential intervals for individual studies, while diamond indicate the pooled effect size. PTSD, posttraumatic stress disorder. Christopher et al. [35], Gerson et al. [37], Grupe et al. [38].

Overall, psychological interventions did not significantly alleviate PTSD symptoms (Hedges’s g (95% CI) = 0.581 (−0.003 to 1.164); *p* = 0.051) with significant heterogeneity (I^2^ = 78.51; *p* = 0.01), reflecting a wide variation in treatment efficacy. Compared to waitlist controls, psychological interventions significantly improved PTSD symptoms (Hedges’s g (95% CI) = 0.640 (0.054 to 1.226); *p* = 0.032) with significant heterogeneity (I^2^ = 71.55; *p* = 0.032) (Figure 4). Because only a single study compared psychological interventions with usual care for PTSD symptoms, a pooled effect size could not be calculated; instead, the findings from that study were summarized descriptively. Taken together, while these results indicate a direction of improvement, the fact that the primary pooled effect narrowly missed statistical significance, combined with high heterogeneity, suggests that the evidence for a general beneficial effect on PTSD symptoms remains inconclusive in the overall analysis.

**Figure 5:**
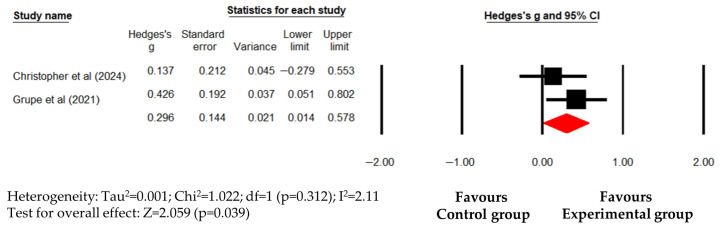
Forest plot showing effect sizes (Hedges’s g) of non-MBIS on PTSD symptoms in police officers. Squares represent effect sizes with 95% confidential intervals for individual studies, while diamond indicate the pooled effect size. PTSD, posttraumatic stress disorder. Christopher et al. [35], Grupe et al. [38].

To investigate this heterogeneity, a subgroup analysis by intervention type was performed. Non-MBIs demonstrated a significant and highly consistent reduction in PTSD symptoms (Hedges’s g (95% CI) = 0.296 (0.014 to 0.578); *p* = 0.036) with minimal heterogeneity (I^2^ = 2.11%; *p* = 0.312) (Figure 5). A pooled effect size for MBIs could not be calculated as only one study met the inclusion criteria. These results indicate that the observed inconsistency in the overall analysis may stem from the inclusion of diverse intervention modalities, with non-MBIs showing a statistically significant trend with notably low heterogeneity. Nevertheless, as the subgroup findings for PTSD are based on a small number of studies, they should be interpreted as preliminary evidence of the potential stability of non-MBI approaches rather than as definitive proof of comparative efficacy.

### 3.5. Risk of Bias and Publication Bias

Among the ten studies, one was classified as having a high risk of bias, while the remaining nine studies were rated as having some concerns (Table 2). The prevalence of ‘some concerns’—primarily stemming from issues in randomization transparency and the inability to blind participants—indicates that the overall certainty of the synthesized evidence should be regarded as moderate to low. Sensitivity analyses were therefore conducted by excluding the high-risk study from all analyses that yielded statistically significant results. After exclusion, all previously significant findings remained robust, with only minimal changes in effect size estimates. Specifically, for mental health outcomes, the overall effect size changed slightly from 0.516 to 0.522; effect sizes compared with usual-care or active control conditions increased from 0.561 to 0.593, and those compared with waitlist controls increased from 0.464 to 0.469. Similarly, for PTSD symptoms compared with waitlist controls, the effect size showed a negligible change from 0.640 to 0.650. These findings indicate that the overall conclusions were not materially influenced by the inclusion of the study with a high risk of bias; nevertheless, the methodological limitations identified in the majority of studies suggest that the precision and reliability of these estimates remain subject to the inherent biases of the primary trials.

**Figure 6:**
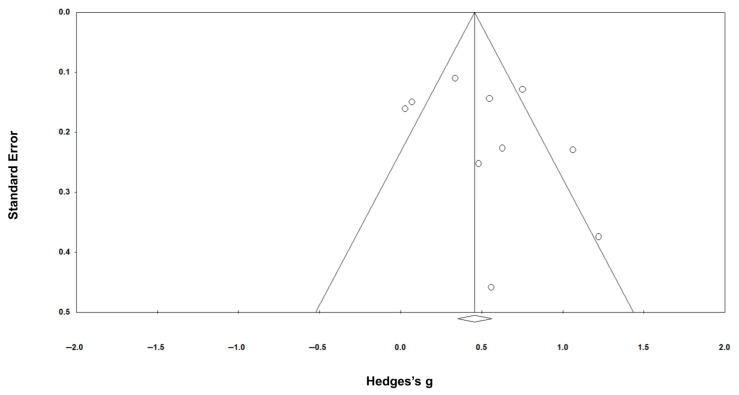
Funnel plot assessing publication bias in studies on the effect of psychological interventions on mental health in police officers. The funnel plot illustrates the relationship between Hedges’s g and their corresponding standard errors for mental health outcomes.

For mental health outcomes, the funnel plot showed significant symmetry (Egger’s intercept = 1.776; *p* = 0.303) (Figure 6). Publication bias was not assessed for subtypes of control conditions because fewer than ten studies were available for each comparison, which precludes reliable evaluation of funnel plot asymmetry. Similarly, publication bias for PTSD symptoms outcomes was not examined, as fewer than ten studies reported PTSD data.

### 3.6. Dose-Response

For mental health outcomes, meta-regression showed no dose–response relationships (β = −0.012; *p* = 0.065), which indicated that, within the range of session hours examined, intervention dose was not a significant moderator of treatment effectiveness. However, these results must be interpreted with caution due to the limited number of studies (*n* = 10), which substantially restricts the statistical power to detect moderator effects. Given the potential for Type II errors under these conditions, this meta-regression should be viewed as an exploratory analysis rather than a definitive exclusion of dose as a relevant factor.

### 3.7. Certainty of Evidence

The certainty of evidence for the primary outcomes was evaluated using the GRADE approach. For general mental health, the certainty was rated as moderate. It was downgraded by one level due to substantial inconsistency (I^2^ = 72.16%). In contrast, the certainty of evidence for PTSD symptoms was rated as low, as it was downgraded by two levels due to both serious inconsistency (I^2^ = 78.51%) and imprecision, reflected in the non-significant overall pooled effect and relatively wide confidence intervals. Although publication bias for PTSD outcomes could not be formally assessed via statistical tests due to the small number of studies, the risk was minimized through a comprehensive search of trial registries and grey literature (Table 3).

## 4. Discussion

The present meta-analysis synthesized evidence from RCTs evaluating the effectiveness of psychological interventions on mental health outcomes among police officers. Overall, psychological interventions demonstrated potential for beneficial effects on general mental health, including depression, anxiety, and stress. While these effects were statistically significant, they were characterized by substantial heterogeneity, as reflected in our GRADE assessment where the certainty of evidence was rated as moderate. This indicates that while the interventions appear generally promising, the strength of this conclusion is bounded by the methodological quality of the primary studies, most of which presented some concerns in risk-of-bias assessments. Consequently, these pooled estimates should be interpreted with caution, as the lack of uniformity suggests that outcomes may vary significantly depending on intervention types and organizational contexts. Furthermore, the generalizability of these findings should be considered within the context of the sampled population, which was predominantly male and primarily situated in Western and South Asian policing structures. In contrast, effects on PTSD symptoms were more variable; although significant improvements were observed in comparisons with waitlist controls, the overall pooled effect across all comparisons did not reach statistical significance. The certainty of evidence for PTSD was rated as low, reflecting both significant inconsistency and imprecision in the estimates. Subgroup analyses indicated that psychological interventions were associated with significant mental health benefits compared with both waitlist and usual-care/active controls, although the latter comparison was based on a smaller number of studies. Furthermore, exploratory subgroup analysis by intervention type revealed that while MBIs showed non-significant trends with high heterogeneity, non-MBIs yielded larger, statistically significant, and more consistent effects. Meta-regression analyses further showed that intervention dose did not significantly moderate treatment effects.

The present findings align closely with previous meta-analyses indicating that psychological interventions may be helpful in reducing psychological distress and PTSD symptoms among first responders [15]. Prior meta-analytic work has reported significant reductions in PTSD, depression, and anxiety across mixed first responder samples, while noting less consistent effects for stress outcomes and substantial heterogeneity [15]. Similarly, Lu and Petersen (2023) identified moderate intervention effects for depression and anxiety but reported non-significant findings for stress, which they attributed to high heterogeneity and the inclusion of non-randomized study designs [18]. The present study provides an incremental but meaningful advancement by focusing exclusively on RCTs and refining the source of this inconsistency. In contrast, the present police-specific analysis demonstrated significant, albeit heterogeneous effects for general mental health outcomes, including depression, anxiety, and stress. This shift in the locus of inconsistency may reflect not only the specific sample composition but also the distinct performance of different intervention modalities. While prior reviews highlighted general variability, our findings suggest that the observed outcomes in the current literature might be particularly driven by non-MBIs—such as structured resilience and coping skills training—which showed more consistent results in this population. This suggests a possibility that among police officers, general psychological distress may be more uniformly responsive to structured, skills-oriented programs than to mindfulness-based approaches, which showed higher variability.

Conversely, PTSD represents a trauma-specific and often chronic condition that may require more intensive, trauma-focused treatments, and many included trials did not specifically target officers with clinically diagnosed PTSD [42], potentially attenuating pooled effects. From a theoretical perspective, the constrained effect on PTSD symptoms might be linked to trauma-related dissociation, which can act as a barrier to effective trauma processing. In the context of policing, chronic exposure to critical incidents may contribute to dissociative responses that complicate standard cognitive-emotional integration [5,6]. It has been hypothesized that interventions failing to address these dissociative barriers may result in less robust recovery of trauma-specific symptoms, even when general distress improves [7]. While our findings regarding non-MBIs show a significant trend in reducing PTSD symptoms, the limited number of studies—particularly the presence of only a single MBI study for this outcome—precludes definitive conclusions. Taken together, these comparisons suggest that while psychological interventions show a promising trend in improving general mental health outcomes across policing contexts, their effects on PTSD symptoms appear highly sensitive to sample characteristics, intervention specificity, and study design, underscoring the importance of police-specific and trauma-focused methodological approaches in future research.

The observed benefits of psychological interventions may be interpreted in light of several theoretically supported mechanisms. Chronic exposure to stress in policing contributes to physiological and affective dysregulation [36,38,43]. The interventions reviewed here—including mindfulness, CBT, and eclectic strategies—share components aimed at enhancing autonomic regulation and cognitive-emotional processing [44,45]. Beyond these standard frameworks, some contemporary trauma models suggest that prioritizing a secure therapeutic alliance may be essential for addressing more complex trauma responses [46]. While these theoretical considerations provide a plausible framework for our findings, we acknowledge that such interpretations remain speculative and should be further validated through targeted empirical research.

A notable finding in this study is that the effect sizes did not differ substantially between comparisons with waitlist versus usual-care/active control groups. However, this interpretation requires nuance; the apparent broad effectiveness may be partly attributed to the specific nature of these control conditions. Several mechanisms likely explain this convergence. Police officers typically experience high baseline stress, and waitlist groups rarely show spontaneous improvement due to ongoing exposure to organizational and operational stressors [2,18,19]. Thus, intervention–waitlist contrasts remain consistently large. Moreover, usual-care/active controls provide only modest therapeutic benefit and lack the depth needed to modify cognitive–emotional and physiological mechanisms targeted by structured psychological interventions [2,18,19]. Therefore, both waitlist and usual-care/active controls produce similar comparative effect sizes because the intervention gains substantially outweigh improvements in either type of control group. These findings reinforce the unique benefits of structured interventions; yet, they also suggest that the magnitude of effect may be context-dependent, particularly in environments where robust active supports are absent.

The findings have potential implications for practice and theory. From a practical perspective, the observed improvements in depression, anxiety, and stress suggest that psychological interventions—particularly structured, skills-based programs focusing on resilience and CBT techniques—could be considered for integration into organizational mental health strategies. Future organizational strategies might benefit from moving beyond mere symptom mitigation toward fostering broader psychological resilience and professional reintegration [8]. Critically, individual-level interventions do not operate in a vacuum; their success is inextricably linked to organizational determinants. Systemic stressor such as high workloads, lack of organizational justice, and a stigmatizing culture can act as barriers that attenuate the effectiveness of even evidence-based programs. Therefore, sustainable mental health benefits require integrating psychological supports into a comprehensive framework that concurrently addresses these structural factors and foster supportive leadership.

While MBIs are popular, their high heterogeneity suggests that their effectiveness may be more sensitive to delivery context. In contrast, while our findings indicated that non-MBI programs appear to offer relatively more stable outcomes in this specific population, these results should be interpreted as preliminary and not as definitive endorsement of one modality over another. The perceived superiority of certain structured programs remains tentative until validated by larger, more diverse trials. Therefore, clinical recommendations regarding specific intervention types should be made with caution, prioritizing a balanced, multi-modal approach that ensures a fit between the intervention components and the unique organizational needs of the policing environment. The findings regarding PTSD are particularly relevant for trauma-prone occupations like policing, where even subthreshold symptoms—which did not reach statistical significance in our overall analysis—can lead to meaningful functional impairment and adverse outcomes [47]. Theoretically, the findings support models emphasizing cognitive appraisal and emotional regulation, though we emphasize that these interpretations must remain tentative, given the moderate-to-low certainty of the evidence and the inherent variability of real-world policing environments.

Nevertheless, several limitations should be acknowledged. First, the meta-analysis was based on a relatively small number of RCTs with a limited total number of participants, which may affect the overall statistical power and generalizability of the findings. Notably, several trials featured small sample size, which restricts the precision of pooled estimates and necessitates caution when interpreting effect sizes. This was particularly for PTSD symptoms. Additionally, the use of a composite general mental health outcome was necessitated by the limited number of studies providing disaggregated data for each specific symptom. While this approach is theoretically supported by the tripartite model, the lack of sufficient primary data prevented a more granular meta-analytic decomposition of these individual domains. Second, there is a lack of long-term follow-up data across the included studies, making it difficult to determine whether the observed beneficial effects are sustained over time within the high-stress policing environment. Third, although we conducted an extensive search, potential publication bias and selective reporting cannot be entirely ruled out, especially given that many included trials were not pre-registered in clinical registries. Fourth, the majority of the interventions were multi-component in nature (e.g., combining psychoeducation, relaxation, and cognitive strategies), which poses significant challenges in isolating the specific active components responsible for the observed improvements. Fifth, while we categorized interventions into MBIs and non-MBIs to explore sources of heterogeneity, this subgroup analysis remains post hoc and hypothesis-generating. Due to the limited number of studies—including only a single MBI study for certain outcomes—these comparative findings should be interpreted with caution and not as definitive evidence of comparative superiority. Furthermore, the predominance of waitlist control conditions restricts generalizability to real-world implementation settings, where officers may have access to alternative active interventions or organizational supports. Additionally, the use of diverse psychometric scales introduced outcome measurement heterogeneity, while the reliance on self-report instruments may have led to response bias or symptom underreporting—a common issue in policing culture. Lastly, limited reporting on instructor training, implementation fidelity, and organizational context prevented a more nuanced examination of contextual moderators that may influence intervention effectiveness. Future research should prioritize adequately powered RCTs with active comparators and granular symptom-level reporting to further clarify the mechanisms underlying differential effects on general mental health and trauma-specific outcome. To enhance the robustness of the evidence base, studies must incorporate longer follow-up periods and provide standardized reporting of intervention content and fidelity. In addition, moving beyond self-report measures by incorporating clinician-rated assessments or objective physiological (e.g., cortisol, heart rate variability) and cognitive (e.g., attentional bias) markers may further clarify the mechanisms underlying differential effects on general mental health and trauma-specific outcomes.

## 5. Conclusions

This systematic review and meta-analysis provide evidence that psychological interventions may produce moderate improvements in general mental health outcomes among police officers, although substantial heterogeneity and the limited number of trials necessitate a cautious interpretation. The findings highlight that non-MBIs, such as resilience and coping skills training, may offer relatively more consistent benefits than MBIs approaches in this specific population. However, the evidence for PTSD symptoms remains limited and of low certainty, with several estimates showing only borderline statistical significance. In conclusion, while structured psychological programs provide added value beyond general wellness supports, their efficacy is notably sensitive to the complex interplay between chronic occupational stress and trauma-related dissociation. The observed variability underscores that traditional interventions may be insufficient to penetrate the dissociative barriers and cognitive fragmentation often found in law enforcement populations. To achieve robust recovery, future strategies for police and first responders should evolve toward integrative, trauma-focused frameworks—such as those emphasizing strong therapeutic alliances and self-actualization—that explicitly address dissociative detachment. Ultimately, moving beyond mere symptom reduction toward fostering holistic existential growth will be essential for the long-term psychological well-being of those serving on the front lines.

## Figures and Tables

**Table 1:** Characteristics of the studies included in the systematic review and meta-analysis.

Authors (Country)	Sample Type	N (Female %)	Age (Year)	Intervention	Session	Comparison	Outcome Measure
Arnetz et al. (2009) [32] (United States)	Active-duty police	E: 9 C: 9 (0%)	NR	Imagery training (Non-MBIs): Psychoeducation including relaxation training, use of guided imagery to facilitate imaginal stressful incidents and mental practices	10 (20 h)	Active control: Critical incident simulation training	Stress: VAS
Chitra et al. (2021) [33] (India)	Active-duty police	EG = 33 CG = 30 (100%)	27.4	CBT (Non-MBIs): CBT-based resilience training including self-awareness, positive attitude development, emotional management (cognitive restructuring, mindfulness), and interpersonal skills	20 sessions (30 h)	Waitlist	Stress: OSI
Chongruksa et al. (2012) [34] (Thailand)	Active-duty police	EG = 7 CG = 9 (0%)	35.6	Escletic group counseling (Non-MBIs): Integrative intervention combining the interactive CBT model (ABC), mindfulness (breathing techniques), art therapy (mandalas), religious interventions (Law of Karma), and reality Therapy for cognitive restructuring and goal achievement.	12 sessions (18~24 h)	Usual care: Psychoeducation	Depression: BDI, SCL-90 Anxiety: SCL-90
Christopher et al. (2024) [35] (United States)	Active-duty police	EG = 31 CG1 = 18 CG2 = 15 (29.8%)	38.1	MBRT (MBIs): Online program adapted from MBRP integrating mindfulness practices (body scan, meditation, movement), CBT, and psychoeducation to target stress resilience and reactivity to police-specific stressors.	8 week (16 h)	Active control: Stress management training Waitlist	Depression: PROMIS-D Stress: PSS-10 PTSD: PCL-5
Christopher et al. (2018) [36] (United States)	Active-duty police	EG = 24 CG = 25 (10.0%)	43.9	MBSR (MBIs): MBSR program specifically for police culture, incorporating practices such as body scan, sitting meditation, and yoga	8 sessions (20 h)	Waitlist	Depression: PROMIS-D Stress: PSQ Anxiety: PROMIS-A
Gersons et al. (2000) [37] (Netherlands)	Active-duty police	EG = 22 CG = 20 (11.9%)	36.5	Brief Eclectic Psychotherapy (Non-MBIs): Manualized individual intervention integrating cognitive-behavioral and psychodynamic approaches. Key components include psychoeducation, imaginary guidance (reliving trauma), writing assignments with mementos, meaning integration, and a farewell ritual.	16 sessions (16 h)	Waitlist	Depression: SCL-90 Anxiety: SCL-90 PTSD: SI-PTSD
Grupe et al. (2021) [38] (United States)	Active-duty police	EG = 54 CG = 56 (41.0%)	NR	MBRT (MBIs): MBSR programs integrating didactic instruction, embodied practices (body scan, movement, compassion), and group inquiry. Specifically tailored to police culture with shorter homework assignments (9–20 min/day) and informal practices applied to operational stressors	8 sessions (16 h)	Waitlist	Depression: PROMIS-D Anxiety: PROMIS-A Stress: PSQ PTSD: PCL-5
Ireland, Malouff, & Byrne (2007) [39] (Australia)	Active-duty police	EG = 28 CG = 39 (41.7%)	38.8	Writing intervention (Non-MBIs): A short-term expressive writing intervention. Officers write about strong emotions (positive or negative) and related action plans in a self-directed, private manner.	4 sessions (1 h)	Waitlist	Depression, Anxiety, Stress: DASS
Ranta & Sud (2008) [40] (India)	Active-duty police	EG = 40 CG = 40 (0%)	NR	Multidimensional intervention (Non-MBIs): Multimodal program integrating stress management (relaxation training), self-management, and mood management techniques. It includes homework assignments and the rehearsal of coping skills in imaginary situations during meditation	3 sessions (3 h)	Usual care: Psychoeducation	Stress: PSQ
Trombka et al. (2021) [41] (Brazil)	Active-duty police	EG = 66 CG = 62 (25.3%)	42.2	Mindfulness (MBIs): Mindfulness-based intervention focusing on universal vulnerabilities using informal practices, radical acceptance, values clarification, and positive psychology, incorporating compassion training and psychoeducation	8 sessions (not specified)	Waitlist	Depression: HADS Anxiety: HADS

BDI: Beck Depression Inventory; HADS: Hospital Anxiety and Depression; PCL-5: Posttraumatic Stress Disorder Checklist for DSM-5; PROMIS-A: OSI: Occupational Stress Inventory; Patient-Reported Outcomes Measurement Information System-Anxiety; PROMIS-D: Patient-Reported Outcomes Measurement Information System-Depression, PSS-10: Perceived Stress Scale-10; SCL-90: Symptom Checklist-90; PSQ: Police Stress Questionnaire; VAS: Visual Analogue Scale; SI-PTSD: Structured Interview for posttraumatic stress disorder.

**Table 2:** Risk of bias in the included studies.

Authors	D1	D2	D3	D4	D5	Overall Risk of Bias
Arnetz et al. (2009) [32]	Some concerns	Low risk	High risk	Some concerns	Some concerns	High risk
Chitra et al. (2021) [33]	Some concerns	Low risk	Low risk	Some concerns	Some concerns	Some concerns
Chongruksa et al. (2012) [34]	Some concerns	Low risk	Some concerns	Some concerns	Some concerns	Some concerns
Christopher et al. (2024) [35]	Low risk	Low risk	Some concerns	Some concerns	Low risk	Some concerns
Christopher et al. (2018) [36]	Low risk	Low risk	Some concerns	Some concerns	Low risk	Some concerns
Gersons et al. (2000) [37]	Some concerns	Low risk	Low risk	Some concerns	Some concerns	Some concerns
Grupe et al. (2021) [38]	Low risk	Low risk	Low risk	Some concerns	Some concerns	Some concerns
Ireland, Malouff, & Byrne (2007) [39]	Some concerns	Low risk	Low risk	Some concerns	Some concerns	Some concerns
Ranta & Sud (2008) [40]	Some concerns	Some concerns	Low risk	Some concerns	Some concerns	Some concerns
Trombka et al. (2021) [41]	Low risk	Low risk	Some concerns	Some concerns	Low risk	Some concerns

**Table 3:** Certainty of evidence of outcomes.

Outcomes	Risk of Bias	Inconsistency	Indirectness	Imprecision	Publication Bias	Certainty of Evidence
Mental health	Not serious	Serious	Not serious	Not serious	Not serious	Moderate
PTSD symptoms	Not serious	Serious	Not serious	Serious	Not serious	Low

## Data Availability

The data presented in this study are available on request from the corresponding author due to the need to ensure that the complex meta-analytic datasets and coding schemes are interpreted within the appropriate methodological context and to prevent potential misinterpretation of the synthesized data.

## Supplementary Materials

The following supporting information can be downloaded at: https://www.mdpi.com/article/10.3390/healthcare14081025/s1, Table S1: PRISMA 2020 checklist.
